# SARS-CoV-2 N Protein Targets TRIM25-Mediated RIG-I Activation to Suppress Innate Immunity

**DOI:** 10.3390/v13081439

**Published:** 2021-07-23

**Authors:** Gianni Gori Savellini, Gabriele Anichini, Claudia Gandolfo, Maria Grazia Cusi

**Affiliations:** 1 Department of Medical Biotechnologies, University of Siena, 53100 Siena, Italy; gabriele.anichini@student.unisi.it (G.A.); claudia.gandolfo@unisi.it (C.G.); mariagrazia.cusi@unisi.it (M.G.C.); 2 “S. Maria delle Scotte” Hospital, Viale Bracci, 1, 53100 Siena, Italy

**Keywords:** innate immunity, SARS-CoV-2 N protein, RIG-I activation

## Abstract

A weak production of INF-β along with an exacerbated release of pro-inflammatory cytokines have been reported during infection by the novel SARS-CoV-2 virus. SARS-CoV-2 encodes several proteins able to counteract the host immune system, which is believed to be one of the most important features contributing to the viral pathogenesis and development of a severe clinical picture. Previous reports have demonstrated that SARS-CoV-2 N protein, along with some non-structural and accessory proteins, efficiently suppresses INF-β production by interacting with RIG-I, an important pattern recognition receptor (PRR) involved in the recognition of pathogen-derived molecules. In the present study, we better characterized the mechanism by which the SARS-CoV-2 N counteracts INF-β secretion and affects RIG-I signaling pathways. In detail, when the N protein was ectopically expressed, we noted a marked decrease in TRIM25-mediated RIG-I activation. The capability of the N protein to bind to, and probably mask, TRIM25 could be the consequence of its antagonistic activity. Furthermore, this interaction occurred at the SPRY domain of TRIM25, harboring the RNA-binding activity necessary for TRIM25 self-activation. Here, we describe new findings regarding the interplay between SARS-CoV-2 and the IFN system, filling some gaps for a better understanding of the molecular mechanisms affecting the innate immune response in COVID-19.

## 1. Introduction

Coronaviruses (CoVs) are a large family of single-stranded, positive-sense RNA viruses that belong to the family *Coronaviridae* [1,2]. Coronavirus has a large RNA genome consisting of the open reading frames (ORFs) 1a and 1b encoding for two polyproteins which are proteolytically cleaved into 16 nonstructural proteins (nsp1–16) that play pivotal roles in the life cycle of CoVs [3,4,5,6]. Almost one-third of the viral genome consists of genes encoding structural proteins, including spike (S), envelope (E), membrane (M), and nucleocapsid (N) proteins. Additionally, the CoVs contain accessory proteins encoded by ORFs located at the 3′ end of their genome [5,6]. Coronaviruses can infect humans and animals (bats, mice and birds) [7,8]; among them, CoV-229E, CoV-OC43, CoV-NL63, CoV-HKU1, SARS-CoV and MERS-CoV have been associated with human diseases [7,8,9,10]. Furthermore, SARS-CoV and MERS-CoV caused severe respiratory illness during the 2002–2003 and 2012 epidemics, respectively [11,12,13,14]. The outbreak of the novel coronavirus SARS-CoV-2, which emerged at the end of 2019, is currently spread worldwide, and the World Health Organization (WHO) declared it a pandemic in March 2020 [15]. SARS-CoV-2 causes coronavirus disease 2019 (COVID-19), which includes a variable spectrum of symptoms, ranging from mild influenza-like syndrome to severe pneumonia, acute respiratory distress and even death [16,17,18,19,20,21,22]. In general, the population that is most affected by COVID-19 are adults, as well as those with pre-existing chronic diseases [23,24]. However, neonates and children can also be infected by SARS-CoV-2 [25]. Many young people or healthy subjects develop asymptomatic or pauci-symptomatic infection with a high risk of human-to-human transmission [26,27,28,29]. SARS-CoV-2 infection mainly targets the respiratory tract. Differences in transmissibility and viral shedding suggest that SARS-CoV-2 significantly differs from other epidemic coronaviruses [29,30,31]. SARS-CoV and MERS-CoV have tropism for the lower respiratory tract, whereas the emergent SARS-CoV-2 virus presents with high viral load in the upper respiratory tract [30,31]. The type I interferon-mediated immune response represents the first line of host defense against viral infection [32]. Viral pathogen-associated molecular patterns (PAMPs), such as viral mRNA and dsRNAs, are produced during early virus infection/replication and recognized by pattern recognition receptors (PRPs), including retinoic-acid inducible gene-I (RIG-I), melanoma differentiation-associated protein 5 (MDA-5) and Toll-like receptors (TLRs) [33]. Both RIG-I and MDA-5 contain two N-terminal caspase activation and recruitment domains (CARDs) that enable interaction with the mitochondrial adaptor protein MAVS [34,35,36,37,38,39,40,41,42] which, in turn, triggers the activation of transcription factors (IRF-3/7; NF-kB and AP-1) involved in IFN expression [34,35,36,37]. Ubiquitination by TRIM25 is a well-characterized regulatory mechanism of RIG-I [36,37,38,39,40,41,42]: the delivery of K63-linked ubiquitin moiety by TRIM25 to the lysine residue at position 172 on RIG-I N-terminal first CARD domain (RIG-IN) results in a marked increase in RIG-I downstream signaling activity [38,39]. Moreover, recent data have demonstrated that unanchored K63 poly-ubiquitin chains play a fundamental role in the full stabilization and activation of the RIG-I tetramer [42]. In order to overcome this first-line defense implemented by the host, viruses have evolved by expressing protein(s) able to block IFN-β production and its downstream activity at different steps in the signaling cascade. 

SARS-CoV and MERS-CoV are known to produce several proteins with antagonistic properties on the interferon signaling. Papain-like protease (PLPro), ORF8b, the nucleocapsid (N), and the ORF3b proteins are well-characterized interferon antagonists [43,44,45,46,47,48,49,50,51].

The SARS-CoV N protein was found to abrogate the activity of the transcription factors IRF3 and NF-κB, resulting in the inhibition of IFN-β synthesis. Furthermore, the antagonistic nature of SARS-CoV N was achieved by hindering RIG-I ubiquitination and activation by TRIM25 [44,45]. A similar mechanism was also described for MERS-CoV [49], suggesting that the SARS-CoV N protein may target multiple factors to overcome the host immune system [50,51]. Different factors are involved in the induction of IFN-β production; therefore, they may act independently during different stages of the viral life cycle, rendering the antagonistic activity of the N protein indispensable as its structural activity in virus replication context. As in other coronaviruses, the nucleocapsid protein is one of the most crucial structural components of the SARS-CoV-2 virus. Hence, major attention has been focused on the characterization of this protein. Studies conducted by other groups have elucidated the IFN-β antagonistic function of this protein [52,53].

In COVID-19 infection, a low induction of type I interferons was detected in the peripheral blood or lungs of patients with a severe clinical picture [54,55,56]. Thus, the expression of SARS-CoV-2 proteins with suppressive activity on innate immunity was analyzed, and a detailed study evidenced the antagonistic properties of some viral proteins [57,58,59,60,61,62]. More details are still needed in order to understand the molecular mechanism residing on SARS-CoV2 escape from the host immune system. For this reason, this study focused on the investigation of the mechanism by which the nucleoprotein N of SARS-CoV2 circumvents IFN-α/β secretion.

## 2. Materials and Methods

### 2.1. Cells, Virus and Chemicals

Human embryonic kidney Lenti-X 293T cells (Clontech, Milan, Italy) were cultured in Dulbecco’s modified Eagle’s medium (DMEM) (Lonza, Milan, Italy) supplemented with 100 U/mL penicillin/streptomycin (Hyclone Europe, Milan, Italy) and 10% heat-inactivated fetal calf serum (FCS) (Lonza, Basel, Switzerland), respectively, at 37 °C. SARS-CoV-2 was isolated on Vero E6 cells (ATCC CRL-1586) from a clinical specimen in the Virology laboratory of “S. Maria alle Scotte” Hospital in Siena, Italy. Transfections were performed using the jetPRIME Transfection Reagent (Polyplus, Illkirch, France). The proteasome inhibitor MG-132 was purchased from Merck Millipore.

### 2.2. Plasmids

Viral RNA was extracted from Vero E6 supernatant of SARS-CoV-2-infected cells using a QIAamp viral RNA mini kit (Qiagen, Hilden, Germany). The N (nt 28218-29477) coding gene was amplified by reverse transcriptase (RT)-polymerase chain reaction (PCR) from purified viral RNA with the following primers (Sigma-Aldrich, Milan, Italy): N BamHI sense 5′-CGCGGACCCCTCTGATAATGGACCCCAAAAT-3′ and N EcoRV antisense 5′-CCGGATATCTGATTAGGCCTGAGTTGAGTCAGC-3′, whereas the HA-tag containing TRIM25 SPRY domain was generated by PCR using the forward 5′-CCGCTCGAGAAGGTGCTGGAGACCTTCCTG-3′ and reverse 5′-ATAGTTTAGCGGCCGCCTAAGCGTAATCTGGAACATCGTATGGGTACTTGGGGGAGCAGA-3′ primers. The reaction was carried out using the SuperScript III One-Step RT-PCR with Platinum Taq (Life Technologies, Milan, Italy) by one cycle of reverse transcription at 55 °C for 30 min and 94 °C for 5 min followed by 40 cycles of PCR (1 min at 94 °C; 30 s at 60 °C; 1 min at 68 °C). The gene was cloned in pcDNA4HisMax-C plasmid (Life Technologies) in frame with the 6xHis tag or in pEF-Bos. The correct sequence of the constructs was confirmed by sequencing (Eurofins Genomics, Milan, Italy). The reporter plasmid encoding Firefly Luciferase downstream of the complete interferon-beta promoter (p125-Luc), the minimal interferon-beta promoter (p55A2) and the plasmids coding the FLAG-tagged RIG-I CARD domains (RIG-IN) were kindly provided by Prof. Takashi Fujita (Tokyo Metropolitan Institute of Medical Science, Tokyo, Japan). The FLAG-tagged human RIG-I and MDA-5 expression plasmids were kindly provided by Prof. A. García-Sastre (Mount Sinai School of Medicine, New York, NY, USA). The plasmids expressing human IRF3 and HA-tagged TRIM25 were kindly provided by L. Martinez-Sobrido (University of Rochester Medical Center School of Medicine and Dentistry, Rochester, NY, USA) and Hiroyuki Oshiumi (Graduate School of Medical Sciences, Kumamoto University, Kumamoto, Japan), respectively. The Renilla Luciferase reporter plasmid was purchased from Promega (Promega, Milan, Italy); HA-tagged K63-only ubiquitin mutant-expressing plasmid was from Addgene (Cambridge, MA, USA). 

### 2.3. Transfection and Immunoblotting

Transient expression of the recombinant proteins was carried out using jetPRIME Transfection Reagent (Polyplus, Illkirch, France) following the manufacturer’s instructions. Lenti-X 293T cells were seeded in a 6-well culture plate at a density of 1 × 10^6^ cells per well and incubated at 37 °C in a 5% CO_2_ atmosphere. The next day, cell monolayers were transfected with selected plasmids and cells were collected at 48 h post-transfection for protein analysis. Where necessary, protein concentration of the whole cell lysates was determined by BCA assay (Pierce, Milan, Italy). Fifty micrograms of total proteins were loaded on SDS-PAGE and, after being transferred to a NitroBind nitrocellulose membrane (Santa Cruz Biotechnology, Heidelberg, Germany), protein expression was detected using anti-Flag M2 (Merck-Millipore) (1:2000), anti-SARS-CoV-2 N rabbit polyclonal antibody (Life Technologies) (1:1000), anti-HA monoclonal antibody (Sigma-Aldrich, St. Louis, MO, USA) (1:1000) or anti-actin (BioLegend, London, UK) (1:500) as loading controls. Horseradish peroxidase (HRP)-conjugated secondary antibody (Merck-Millipore) (1:5000) was used as a secondary antibody. Quantitative comparisons among samples were performed by densitometric analysis using ImageJ software.

### 2.4. Co-Immunoprecipitations (Co-IP)

Lenti-X 293T cells were plated in 9 cm Petri dishes and transfected with 1 µg of RIG-I-expressing plasmid along with 0.2 µg of both TRIM25- and HA-ubiquitin K63-only-expressing plasmids. For protein ubiquitination detection, 36 h post-transfection exhausted medium was replaced with fresh DMEM supplemented with 1 μM of MG-132 proteasome inhibitor. Samples were collected after additional 12 h. For immuno-precipitations, cell lysates were prepared in RIPA buffer (50 mM TrisHCl (pH 7.5); 150 mN NaCl; 1 mM EDTA; 1% Triton X-100) supplemented with a cOmplete anti-protease cocktail (Roche, Milan, Italy). Cleared lysates were incubated overnight at 4 °C with anti-FLAG M2 magnetic beads with gentle rotation. For RIG-I ubiquitination detection, denaturing immuno-precipitations were performed. Cell samples were lysed in 1% SDS by boiling for 10 min. Then, SDS was diluted to 0.1% final concentration with RIPA buffer supplemented with the cOmplete anti-protease cocktail (Roche, Milan, Italy). Proteins were captured with anti-FLAG M2 magnetic beads overnight at 4 °C, after which they were collected using a magnetic stand and extensively washed with phosphate buffered-solution (PBS). Bound proteins were eluted by boiling with 2× denaturing sample buffer, loaded on SDS-PAGE and probed by immunoblotting, as described above. 

### 2.5. Luciferase Reporter Assay

For analyses, 2 × 10^5^ Lenti-X 293T cells were seeded in 24-well plates and transfected with indicated plasmids, as previously described. Briefly, 0.2 µg of p125-FFLuc, 0.05 μg of RIG-I, MDA-5 or RIG-IN and, where indicated, 0.5 μg of N expressing plasmid were co-transfected. Where indicated, 50 ng of HA-TRIM25, HA-Riplet or hIRF3 plasmids were co-transfected. Empty plasmids were used to normalize the total amount of DNA. Twenty nanograms of pSV40-RL were co-transfected as internal controls. Thirty-six hours post-transfection, cells were stimulated by transfection with 2 μg/well of poly (I:C). After an additional 12 h, cells were collected and luciferase activities were measured on lysates using the Dual Luciferase reporter assay reagent (Promega, Madison, WI, USA), according to the manufacturer’s instructions. Results are given as mean values of several experiments ± standard deviations (SD).

### 2.6. Statistics

The mean differences were statistically analyzed using GraphPad Prism 6 (GraphPad Software San Diego, CA, USA) by means of the Fisher test to compare prevalence rates among different study groups. Statistical significance was set at *p* < 0.05, and was two-tailed.

## 3. Results

### 3.1. SARS-CoV-2 N Protein Specifically Impairs RIG-I Signaling Pathway

**Figure 1:**
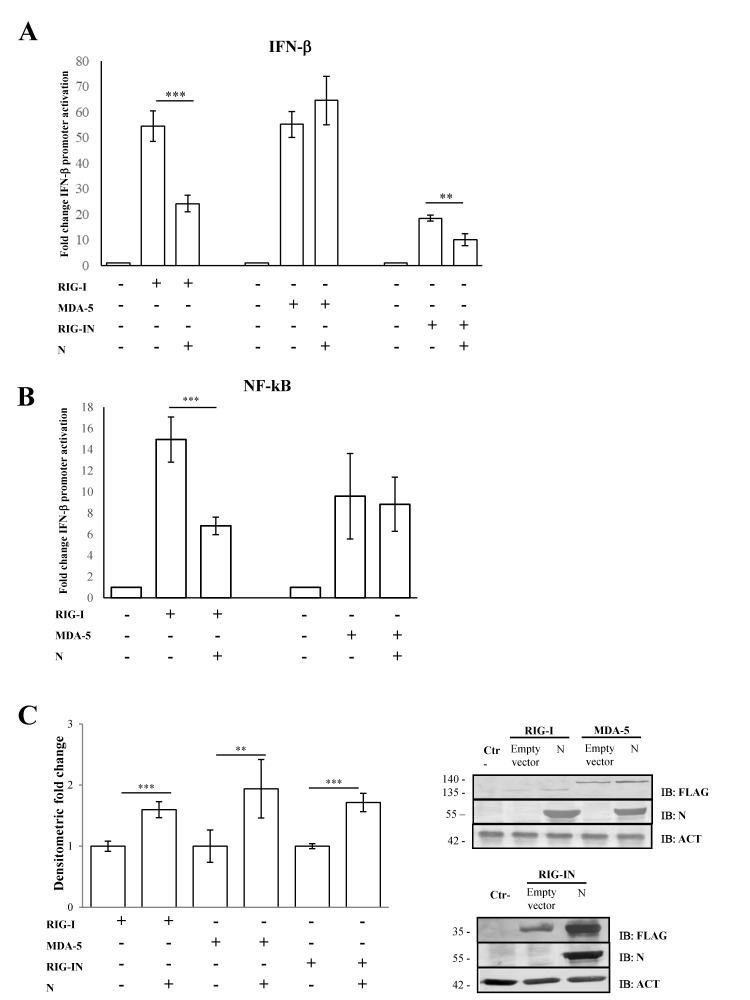
SARS-CoV-2 N counteracts RIG-I-mediated signaling. (**A**) SARS-CoV-2 N protein inhibited IFN-β promoter activation. Lenti-X 293T cells were co-transfected with the IFN-β Firefly luciferase reporter plasmid, RIG-I-, RIG-IN- or MDA-5-expressing plasmids, with or without the N-expressing plasmid. Renilla luciferase control reporter plasmid was included as an internal control for further normalization. Firefly luciferase activities were normalized with respect to Renilla luciferase values, and fold induction was estimated with respect to the relative mock-treated sample. (**B**) The inhibitory effect of SARS-CoV-2 N protein on the minimal IFN-β promoter containing the NF-κB responsive element was assessed by a luciferase reporter assay, as described above. (**C**) Fifty micrograms of whole cell lysates (WCLs) were further analyzed by immunoblotting for RIG-I, RIG-IN, MDA-5 and N proteins using anti-FLAG M2 and anti-N monoclonal antibodies. Loading control was represented by the immuno-detection of actin protein. Densitometric analyses of reactive bands were performed with ImageJ software. Results are plotted as the mean fold change in RIG-I, RIG-IN or MDA-5 with respect to relative actin levels from (*n* = 3) independent experiments ± SD. Significance was determined with *p*-values (*** *p* < 0.005, 0.005 < ** *p* < 0.01).

Previous studies conducted on SARS-CoV-2 demonstrated that the N structural viral protein was able to suppress RIG-I-mediated IFN-β promoter activation, a key sensor of RNA virus infection [52]. A recent publication reported that SARS-CoV-2 virus N protein exhibited such behavior presumably due to its ability to bind RIG-I [53]. Here, we confirmed that SARS-CoV-2 N protein was able to block IFN-β promoter activation in a highly specific manner by inhibiting RIG-I, but not MDA-5, upon poly(I:C) stimulation. Indeed, the transient expression of the N protein in Lenti-X 293T cells affected the luciferase reporter gene expression when RIG-I (*p* = 0.0016) but not MDA-5 (*p* = 0.205) was over-expressed (Figure 1A, Table S1).

Afterwards, we evaluated the N protein activity on RIG-I CARD domains (RIG-IN). RIG-IN, lacking the repressor and helicase domains (RD and CTD) [33], is known to be constantly active in the absence of stimuli; therefore, IFN-β induction inevitably occurs. In this system, a small amount of RIG-IN expression was sufficient to induce IFN-β promoter activation, which showed significant abrogation by the presence of the N protein (Figure 1A, Table S1). In parallel, the antagonistic behavior of the N protein was tested on the minimal IFN-β promoter containing the NF-κB responsive element. Additionally, in this case, the activation of the minimal IFN-β promoter was hindered when RIG-I (*p* = 0.002) was expressed along with the viral protein. In contrast, N protein did not interfere with the MDA-5 signaling pathway (Figure 1B, Table S1). The capability of the N protein to block the NF-κB transcription factor activity confirmed the N antagonistic function mostly on the early stage of the signaling cascade leading to IFN-β production. To address whether the antagonistic properties of the N protein were due to a down-regulation of RIG-I cellular levels, the cytoplasmic amount of RIG-I was evaluated. As shown in Figure 1C, the presence of SARS-CoV-2 N protein did not negatively affect the cellular accumulation of RIG-I, which, in turn, was stabilized by the presence of the viral protein (*p* = 0.004), as reported by semi-quantitative immunoblotting (Figure 1C, Table S1). Likewise, MDA-5 and RIG-IN levels were significantly increased (*p* = 0.01; *p <* 0.0001, respectively) when the N protein was co-expressed (Figure 1C, Table S1).

### 3.2. SARS-CoV-2 Nucleoprotein Prevents RIG-I Activation by TRIM25

**Figure 2:**
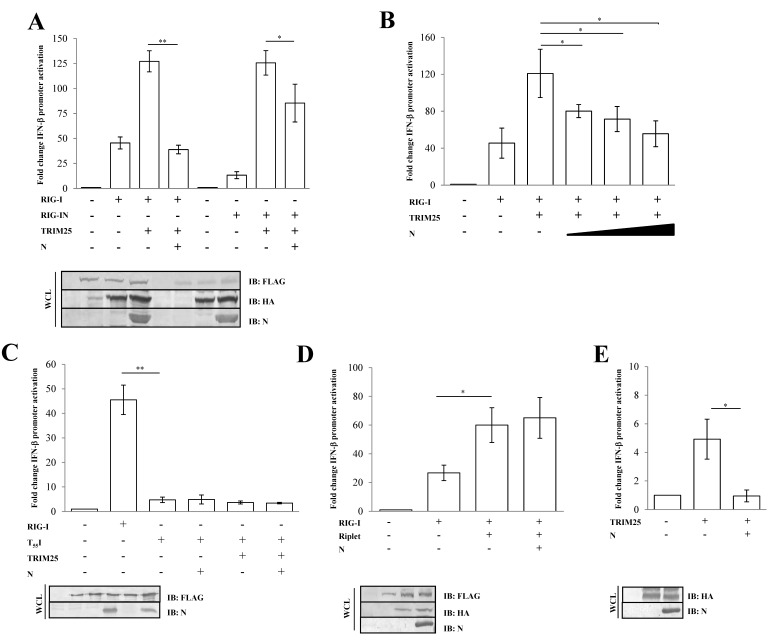
SARS-CoV-2 N protein inhibits TRIM25-mediated RIG-I activation to counteract IFN-β secretion. Lenti-X 293T cells were co-transfected with the IFN-β-Luc Firefly luciferase reporter plasmid and the Renilla luciferase control reporter plasmid along with RIG-I- and TRIM25-expressing plasmids. Where indicated, fixed (**A**) or increasing (**B**) amounts of SARS-CoV-2 N were co-expressed. (**C**) Wild-type RIG-I was replaced by its T_55_I mutated counterpart. Cells were collected at 48 h post-transfection, prior to 12 h of stimulation with poly(I:C). The luciferase activity of the cell lysates was analyzed with the dual luciferase reporter assay system (Promega). Fold changes in IFN-β promoter activation were calculated with respect to the relative reference sample based on Firefly luciferase values normalized to Renilla luciferase activities. (**D**) The specificity of SARS-CoV-2 N on TRIM25 was achieved by enhancing RIG-I activation through the over-expression of the stimulatory protein Riplet or (**E**) solely on TRIM25 expression. Luciferase assays were assessed and the fold change in activation was calculated as previously described. Results were plotted as mean values (*n* = 4) ± standard deviation (SD), and significance was determined by *p*-values 0.005 < ** *p* < 0.01, 0.01 < * *p* < 0.05). Cell lysates from reporter gene assays were analyzed by immunoblotting. Fifty micrograms of WCLs were loaded on SDS-PAGE and probed with specific monoclonal antibodies for protein detection.

The activation of RIG-I is tightly controlled by its ubiquitination by the E3 ligase TRIM25 on lysine residues at the 172-position on the second RIG-I CARD domain [38,39]. To assess whether the N protein inhibitory activity was due to a direct effect on early RIG-I activation or on a downstream step in the cascade, an IFN-β reporter assay was performed in the presence of TRIM25 over-expression. The presence of TRIM25 significantly enhanced RIG-I signaling (2.4-fold induction; *p* = 0.0006) (Figure 2A, Table S2). 

However, the N protein completely abrogated TRIM25-mediated RIG-I activity (0.4-fold decrease; *p* = 0.009, Table S2), as shown in Figure 2A. In a similar manner, the N protein showed its antagonistic properties on RIG-IN activation by TRIM25 (Figure 2A, Table S2). Thereafter, the SARS-CoV-2 N protein substantially attenuated the RIG-I/TRIM25 signaling pathway in a dose-dependent manner, as evidenced by expressing an increasing amount of the protein (Figure 2B, Table S2). Moreover, the antagonistic nature of the viral protein was not detected on the T_55_I RIG-I mutant (*p* = 0.929), which lacks a TRIM25 binding site, confirming a direct effect of the N protein on TRIM25-mediated RIG-I activation rather than on other RIG-I domains (Figure 2C, Table S2). To support these observations, we also evaluated the specific activity of the N protein on Riplet, another E3 ubiquitin ligase involved in the control of RIG-I functions. The N protein was unable to counteract the stimulatory activity of Riplet on RIG-I (Figure 2D, Table S2). To further verify the inhibitory effect of N protein on TRIM25, we tested whether N protein affected endogenous RIG-I activation by transfecting Lenti-X 293T cells with TRIM25 alone. As expected, RIG-I activation was induced upon overexpression of the E3 ubiquitin ligase (*p* = 0.0153), although this induction was counteracted by ectopic expression of the N protein (Figure 2E, Table S2).

### 3.3. IRF3 Activation Is Impaired by N-Mediated RIG-I-TRIM25 Axes Inhibition 

**Figure 3:**
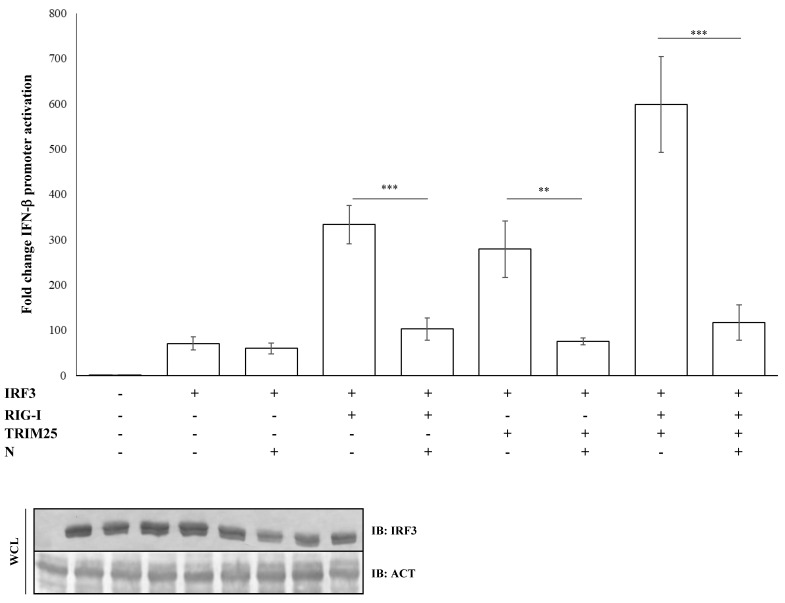
IRF3 activation is impaired due to inhibition of the RIG-I-TRIM25 pathway. Lenti-X 293T cells were co-transfected with the IFN-β-Luc Firefly luciferase reporter plasmid and the Renilla luciferase control reporter plasmid along with the IRF3-expressing plasmid. Where indicated, co-transfections were performed, including expressing plasmids for RIG-I, TRIM25 or SARS-CoV-2 N. Cells, previously stimulated for 12 h with poly(I:C), were collected at 48 h post-transfection. The luciferase activity of the cell lysates was analyzed with the dual luciferase reporter assay system (Promega). Fold changes in IFN-β promoter activation were calculated with respect to the relative reference sample based on Firefly luciferase values normalized to Renilla luciferase activities. Results were plotted as mean values (*n* = 4) ± standard deviation (SD) and significance was determined by *p*-value (*** *p* < 0.005, 0.005 < ** *p* < 0.01). Cell lysates from reporter gene assays were analyzed by immunoblotting.

The nucleocapsid (N) protein of SARS-CoV was shown to inhibit the activation and nuclear translocation of the transcription factor IRF3 induced by poly(I:C), a synthetic double-stranded RNA analogue, thus resulting in a potent antagonist of type-I IFNs. Several studies on SARS-CoV demonstrated that the N protein inhibited IRF3 activation through the early PRR recognition stage of the signaling pathway [44]. Further studies showed that SARS-CoV N protein inhibited IRF3 activity through the suppression of RIG-I ubiquitination/activation by TRIM25 [45]. Regarding SARS-CoV-2, we observed that the IFN-β promoter activation due to IRF3 overexpression was not perturbed by the presence of the N protein (fold decrease 0.85; *p* = 0.373, Table S3). In contrast, the antagonistic function of the viral protein was fully restored when IRF3 activation was induced by RIG-I (fold decrease 0.3; *p* = 0.0012), TRIM25 (fold decrease 0.27; *p* = 0.005, Table S3) or both (fold decrease 0.2; *p* = 0.0018, Table S3) proteins co-expression, confirming the specificity of SARS-CoV-2 N protein for the RIG-I–TRIM25 protein complex (Figure 3). 

### 3.4. The SARS-CoV-2 N Protein Forms a Stable Complex with TRIM25

**Figure 4:**
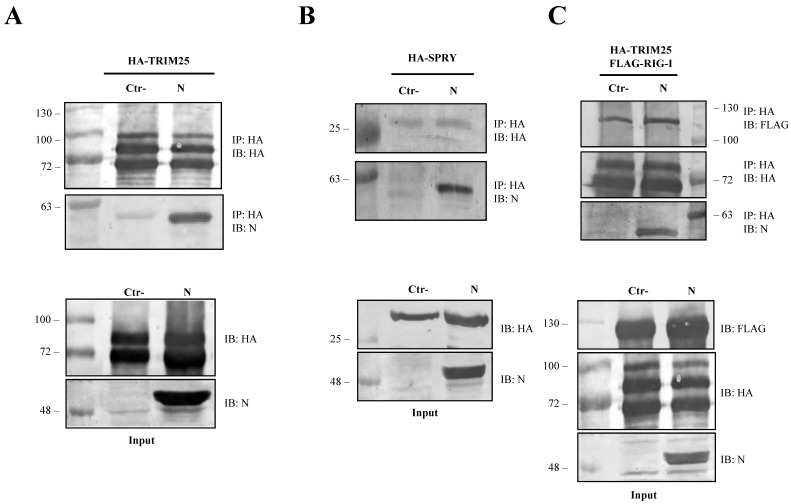
SARS-CoV-2 N protein is an interacting partner of TRIM25. The ability of SARS-CoV-2 N protein to interact with TRIM25 and RIG-I was determined by co-immunoprecipitation (Co-IP) on lysates of transfected Lenti-X 293T cells. (**A**) The formation of a stable immune complex was detected when TRIM25 was transiently over-expressed along with the N protein following Co-IP with anti-HA. (**B**) The N viral protein presented affinity with the SPRY domain of TRIM25 because the viral protein was detected in the anti-HA Co-IP performed on lysates of Lenti-X 293T cells expressing HA-SPRY, alone or in combination with the N protein. (**C**) The capability of SARS-CoV-2 N protein to disturb the RIG-I-TRIM25 interaction was then investigated by Co-IP on lysates of Lenti-X 293T cells simultaneously expressing RIG-I, TRIM25 and N. The interaction between RIG-I and TRIM25 was not affected by the presence of the viral protein. Indeed, their interaction was detected as co-immunoprecipitating with anti-HA antibody in the samples in which the N protein was either present or absent. Blots are representative of (*n* = 3) independent experiments. Fifty micrograms of WCLs were loaded on SDS-PAGE and immunostained as protein expression controls.

To further dissect the involvement of the SARS-CoV-2 N protein on the RIG-I and TRIM25 cascade, lysates of Lenti-X 293T cells exogenously expressing the N protein, along with HA-TRIM25, were extracted with anti-HA antibody. The immunoblotting performed on co-immunoprecipitation complexes revealed that SARS-CoV-2 N protein is associated with TRIM25 because it was readily detected as being associated to the anti-HA precipitated protein (Figure 4A). 

**Figure 5:**
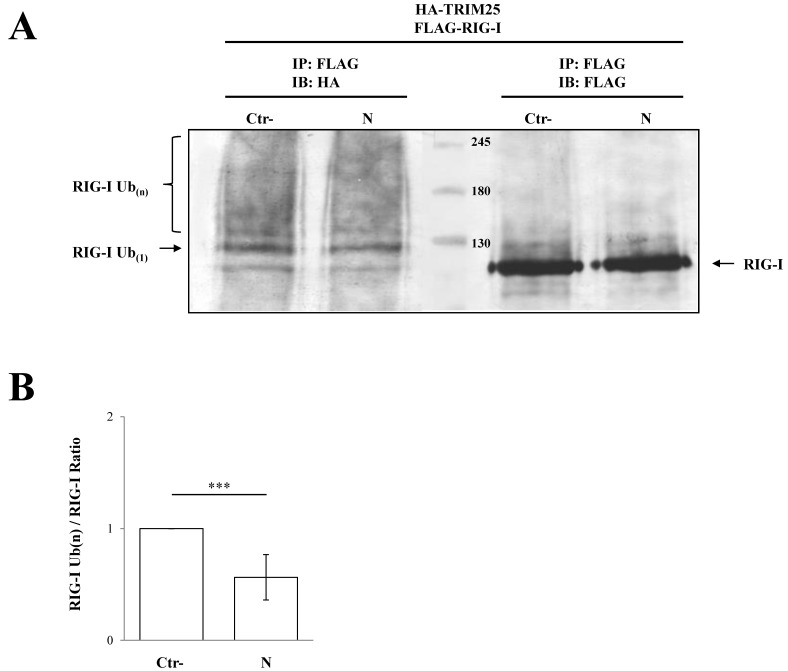
TRIM25 E3 ubiquitin ligase activity was impaired by the presence of SARS-CoV-2 N protein. The transient expression of SARS-CoV-2 N protein affected the ability of TRIM25 to mediate RIG-I ubiquitination in Lenti-X 293T cells. (**A**) Cells transfected with plasmids expressing FLAG-RIG-I, HA-TRIM25, HA-K63-only ubiquitin mutant and, where indicated, the SARS-CoV-2 N protein, were treated with the proteasome inhibitor MG-132 for 12 h. Lysates, prepared under denaturing conditions as described in the Material and Methods section, were immunoprecipitated with anti-FLAG magnetic beads, and precipitated proteins were resolved by SDS-PAGE. Pulled proteins were detected by immunoblotting with anti-HA or anti-FLAG monoclonal antibodies. Blots are representative of at least two biologically independent experiments. (**B**) The RIG-I ubiquitination rate was determined by densitometric analysis calculating the ratio between mono- and poly-ubiquitinated RIG-I (anti-HA) and relative RIG-I levels (anti-FLAG). Fold changes in RIG-I ubiquitination were calculated for N-containing samples relatively to the control sample. Results are expressed as mean (*n* = 3) fold change ± standard deviation (SD), and significance was determined by *p*-value (*** *p* < 0.005).

Co-immunoprecipitation experiments further confirmed that SARS-CoV-2 N protein interacted with the TRIM25 functional domain SPRY. The anti-HA Co-IP performed on cell lysates expressing HA-SPRY and N showed that the interaction between N and TRIM25 occurred at the SPRY sequence of the ubiquitin ligase (Figure 4B). These findings clearly showed that TRIM25 was an association partner of the SARS-CoV-2 N protein in transfected human cells. Next, we hypothesized that TRIM25-RIG-I interaction might be influenced by the presence of SARS-CoV N protein. To corroborate this hypothesis, SARS-CoV-2 N protein was expressed in Lenti-X 293T cells along with FLAG-RIG-I and HA-TRIM25. The interaction profile determined by Co-IP clearly demonstrated that the three proteins formed a multi-complex (Figure 4C). Indeed, immunoblotting performed on the immune-complex of the TRIM25 Co-IP sample evidenced the presence of both RIG-I and N (Figure 4C). Thus, we can speculate that the N protein interposes between RIG-I and TRIM25, which causes depletion and impedes full RIG-I activation. To further support this hypothesis, the level of TRIM25-mediated RIG-I ubiquitination was evaluated. In Lenti-X 293T cells, RIG-I underwent substantial ubiquitination by the overexpression of TRIM25 and K63-only ubiquitin (Figure 5, Table S4). Such ubiquitination was significantly reduced (*p* = 0.006) by the presence of the N protein which, consistently with previous results, reduced RIG-I ubiquitination and activation by TRIM25 without affecting its cellular accumulation (Figure 5, Table S4). 

## 4. Discussion and Conclusions

Antagonism to the host innate immune response by viral proteins is a crucial point in virus replication and could affect the outcome of the infection [32,58,59,60,61,62]. Viral escape from a host innate immune response reflects an efficient and unperturbed viral replication due to the absence of type I interferon secretion [43,44,45,46,47,48,49,50,51,52,53,54,55,56,57,58]. The lack of an adequate IFN-β production and antiviral protein expression may be critical to COVID-19 pathogenesis. SARS-CoV, a virus closely related to SARS-CoV-2, utilizes multiple strategies to antagonize the host antiviral response [43,44,45,46,47,48]. Notably, ORF6 and the nucleoprotein N play a critical role in type I IFN production [44,45]. Notwithstanding, the N protein represents the most important viral antagonistic factor due to its multi-functional activity on the signaling pathway leading to IFN-β production [50]. Based on the similarity between SARS-CoV and SARS-CoV-2, a conserved antagonistic function to the innate immunity for the SARS-CoV-2 N protein has already been described [52,53]. In the present study, we demonstrated a mechanism by which SARS-CoV-2 N protein was able to inhibit IFN-β secretion. Moreover, despite genetic variability among SARS-coronaviruses, the N protein shared the ability to antagonize the host innate immune response, suggesting that this function was evolutionarily conserved and fundamental for virus replication, survival and spread. An in-depth analysis revealed that the SARS-CoV-2 N displayed an IFN-β antagonistic mechanism similar to that of SARS-CoV, because it was involved in this mechanism at a very early step in viral infection, in particular, targeting RIG-I. 

Viral antagonistic proteins can exert their activity at different steps in the RIG-I- and MDA-5-mediated signaling cascades in order to block IFN-β production; therefore, we improved the characterization of the SARS-CoV-2 N inhibitory mechanism by investigating its effects on the PRRs or downstream effectors (IRF3). Although SARS-CoV-2 N protein did not lead to a negative regulation of RIG-I or MDA-5 cellular accumulation, it displayed an IFN-β counteractive mechanism similar to that of SARS-CoV. Indeed, the antagonistic properties of the SARS-CoV-2 N protein were addressed to RIG-I and its CARD domains (RIG-IN), because a significant inhibition of IFN-β promoter activation was evidenced in vitro through the IFN-β promoter reporter assay, when an overexpression of N protein was tested with RIG-I and its CARD domains (RIG-IN). Moreover, this inhibitory function was also confirmed using the minimal IFN-β promoter plasmid, consisting of only the NF-κB responsive element. We demonstrated that the antagonistic property of the SARS-CoV-2 N protein was addressed to the TRIM25-mediated activation of RIG-I. A significant inhibition was noticed when the overexpression of the co-stimulatory protein TRIM25 was associated with the viral N protein. The affinity of the N protein for TRIM25 was clearly demonstrated by co-immunoprecipitation experiments, where the two proteins were detected as associated partners. Similarly to SARS-CoV, this interaction was mapped to the SPRY domain of TRIM25, harboring the catalytic domain and the interface for RIG-I recognition. Further proof of this mechanism came from the ability of N protein to block the ubiquitinating activity of TRIM25 on RIG-I which, however, remained accumulated into the cell. These results led to the hypothesis that TRIM25-RIG-I axis could be altered by the presence of the N protein. Several lines of evidence point toward TRIM25 as a key modulator of RIG-I, although it is one of the multiple host proteins having this function. Among them, Riplet plays a similar role, having the ability to ubiquitinate and control RIG-I signaling. Notwithstanding, SARS-CoV-2 N protein seemed to exert a highly specific action only on TRIM25, because its presence did not affect Riplet activity. The detailed interactions among the RIG-I/TRIM25/N triple complex is currently being investigated [52,53]. In this study, the results suggested that the N protein intervened on the RIG-I and TRIM25 axis by hindering TRIM25, whose E3 ligase activity on RIG-I was suppressed and, consequently, triggered the sequential steps culminating in IFN production. In this context, IRF3 activity was not directly affected by the presence of the N protein. The presently described N protein activity, combined with the ability of several viral proteins (ORF3, ORF6, nsp13 and nsp14) to suppress host innate immunity, establishes a comprehensive suppression of type I IFN production during SARS-CoV-2 viral infection; in particular, suppressing IFN-β by directly antagonizing the enzymatic function of TRIM25. Moreover, these data help to increase our knowledge on immune responses to SARS-CoV2 virus, which represents an important worldwide threat for humanity. 

In the light of recent outbreak of SARS-CoV-2 variants, much attention is focused on mutations occurring in the spike protein, the major concern in vaccination trials. Notwithstanding, conserved mutations among virus variants have also been reported in the N protein [61,62]. The nucleoprotein modulates the innate immune response; therefore, mutations in its sequence can play an important role, rather than in virus infectivity and transmissibility, in determining its antagonistic properties and the clinical outcomes to SARS-CoV-2 infection.

## Data Availability

Not applicable.

## Supplementary Materials

The following are available online at https://www.mdpi.com/article/10.3390/v13081439/s1, Table S1–S4.
